# Pathophysiology of ADHD and associated problems—starting points for NF interventions?

**DOI:** 10.3389/fnhum.2015.00359

**Published:** 2015-06-24

**Authors:** Björn Albrecht, Henrik Uebel-von Sandersleben, Holger Gevensleben, Aribert Rothenberger

**Affiliations:** Department of Child and Adolescent Psychiatry, University Medical Center GöttingenGöttingen, Germany

**Keywords:** Neurofeedback (NF), ADHD, ODD/CD, tic disorder, comorbidity, children, neurobiology

## Abstract

Attention deficit hyperactivity disorder (ADHD) is characterized by severe and age-inappropriate levels of hyperactivity, impulsivity and inattention. ADHD is a heterogeneous disorder, and the majority of patients show comorbid or associated problems from other psychiatric disorders. Also, ADHD is associated with cognitive and motivational problems as well as resting-state abnormalities, associated with impaired brain activity in distinct neuronal networks. This needs to be considered in a multimodal treatment, of which neurofeedback (NF) may be a promising component. During NF, specific brain activity is fed-back using visual or auditory signals, allowing the participants to gain control over these otherwise unaware neuronal processes. NF may be used to directly improve underlying neuronal deficits, and/or to establish more general self-regulatory skills that may be used to compensate behavioral difficulties. The current manuscript describes pathophysiological characteristics of ADHD, heterogeneity of ADHD subtypes and gender differences, as well as frequently associated behavioral problems such as oppositional defiant/conduct or tic disorder. It is discussed how NF may be helpful as a treatment approach within these contexts.

## Introduction

Difficulties with Inattention or Hyperactivity and Impulsivity as the core symptoms of Attention deficit Hyperactivity disorder (ADHD) are a frequent psychosocial burden. With an early onset during childhood, ADHD is often persisting throughout life. It is a heterogeneous disorder, and a challenge to treat. In light of this heterogeneity, the most promising treatment approach should be multimodal in nature (Taylor et al., 2004; Swanson et al., 2008). Pharmacological interventions particularly with stimulants such as methylphenidate and amphetamine sulfate, as well as non-stimulants like Atomoxetine are highly effective in reducing ADHD symptoms (Banaschewski et al., 2006; King et al., 2006), but long-term effectiveness is still questionable (Molina et al., 2009; van de Loo-Neus et al., 2011). In addition, side-effects, non-response and prejudice have raised interest in non-pharmacological treatment options (Sonuga-Barke et al., 2013; Daley et al., 2014).

Neurofeedback (NF) as a non-pharmacological intervention for ADHD utilizes cognitive-behavioral therapeutic elements to gain access on and practice brain activity (Arns et al., 2014). In an operant learning paradigm, specific neural activity is quantified by means of Electro-Encephalography (EEG) or functional Magnetic Resonance Imaging (fMRI) and fed back in real time with an easily accessible optical or acoustic signal. In general, the participants learn to modulate their brain activity towards an* a priori* specified criterion (standard EEG-based NF protocols require the participants to increase beta (13–20 Hz) and to decrease theta (4–8 Hz) activity, or to train slow cortical potentials (SCP) in order to modulate cortical excitability); and successful trials are positively reinforced.

Gevensleben et al. provide two different frameworks, how NF may be effective in ADHD (Gevensleben et al., 2014c). On the one hand, following a “conditioning and repairing model”, NF may be used to compensate specific neurophysiological deficits present in patients with ADHD, which in turn ameliorates impairments. On the other hand, the “skill-acquisition model” suggests that NF may be used to train and enhance self-regulation skills not necessarily impaired in ADHD, but may in turn, by means of active transfer and supportive coaching, be used to compensate existing deficits.

There are a number of studies indicating potential effectiveness of NF on ADHD symptom severity, but further evidence particularly from randomized controlled trials using more blinded assessments is required (Arns and Strehl, 2013; Sonuga-Barke et al., 2013; Micoulaud-Franchi et al., 2014). On the other hand, further double-blinding or particularly sham feedback may diminish motivation and the belief in self-efficacy in both participants receiving sham and NF interventions, and may thus question an important precondition for effective trainings (Logemann et al., 2010; Gevensleben et al., 2012). Further details about NF interventions can be found in a number of reviews and conceptual papers (Strehl et al., 2006; Heinrich et al., 2007; Arns et al., 2014; Gevensleben et al., 2014b), and recent advances in the field are described in this issue on “NF in ADHD”.

The following selective overview describes pathophysiological characteristics of ADHD alongside their potential relevance for NF intervention. First, a brief overview of the clinical characteristics of ADHD is given. Second, pathophysiological characteristics of ADHD linked with difficulties in cognitive functions and motivation as well as during resting state are described, and third, a number of associated problems such as frequent comorbidities of ADHD with Conduct- and Tic-disorders are presented. Finally, perspectives for NF interventions will be considered within these contexts.

## Characteristics of Attention Deficit Hyperactivity Disorder

ADHD is currently considered as a neurodevelopmental disorder. It is characterized by severe and age-inappropriate levels of inattention and hyperactivity/impulsivity that are present in at least two areas of life for over 6 months (WHO, 1993; APA, 2013). According to the fifth edition of the Diagnostic and Statistical Manual (DSM-V), subtypes with predominantly Inattentive or Hyperactive/Impulsive characteristics as well as a combined type are distinguished. In any case, the symptoms must already be manifest in childhood (before age of seven following the DSM-IV, and before age of 12 according to the recently revised DSM-V; Kieling et al., 2010), and must not be better explained by other disorders.

ADHD is one of the most frequent problems in psychiatry. The core symptoms of ADHD are present in approximately 5% of children and adolescents, irrespective of cultural background, and with a strong overrepresentation of boys (Polanczyk and Rohde, 2007). In about one or two out of three of children with ADHD, the symptom may persist with clinical significance into adulthood, leading to a slightly lower prevalence of more than 3% in adults (larger in higher income countries), which makes ADHD a life-long problem for many patients (Fayyad et al., 2007; Polanczyk and Rohde, 2007). Childhood ADHD may lead to lower educational, occupational, social and clinical outcomes in adulthood even if it remits early on, and may thus not be considered as a benign disorder (Klein et al., 2012).

## ADHD and its Neuronal Background

ADHD is associated with a number of neurophysiological deficits. More recent theoretical approaches integrate clinical symptoms and neuropsychological difficulties within a framework of specific brain dysfunctions: cognitive deficits may emerge from dysfunctions particularly in fronto-striatal or meso-cortical brain networks, while problems with reward processing may be associated with dysfunctions in the mesolimbic dopaminergic system (Sagvolden et al., 2005; Sonuga-Barke, 2005). However, deficits in ADHD may already be seen in the resting brain, and a more fundamental neuronal network approach suggests that in ADHD particularly Default-Mode-Network (DMN) activity (usually prominent during rest) may interfere with activity in neuronal networks engaged in task processing, leading to difficulties in state regulation and periodic attentional lapses (Sonuga-Barke and Castellanos, 2007; Castellanos and Proal, 2012).

### Cognitive Functions

There are a number of cognitive theories that describe impairments in executive functions as a central problem in ADHD (Pennington and Ozonoff, 1996; Tannock, 1998; Sergeant, 2000; Biederman, 2005). Several theoretical accounts propose a “top-down” executive system responsible for inhibition, working memory and cognitive flexibility, which is particularly active when more complex demands require adaptation and effortful control (Baddeley and Della Sala, 1996; Miller and Cohen, 2001; Diamond, 2013). Following Barkley, children with ADHD may show a core deficit in behavioral inhibition, which in turn leads to impairments in working memory, self-regulation, internalization of speech and reconstitution (Barkley, 1997). This account has been put forward in the more recent “multiple pathway” models of ADHD, which emphasize besides cognitive deficits also motivational or reward processing problems (Nigg et al., 2005; Sonuga-Barke, 2005).

Cognitive problems in ADHD are reported in numerous studies with different tasks. It was frequently found that children with ADHD display several deficits in tasks that demand executive control, i.e., their reaction-times were slower and more variable, and more errors were made. This has been demonstrated for important aspects of executive functions such as set shifting assessed with the Wisconsin Card-Sorting Task or planning and problem solving as required in the Tower-of-Hanoi paradigms (Barkley et al., 1992; Klorman et al., 1999). On the other hand, interference control during Stroop- or Simon tasks yielded mixed or even negative results, particularly when confounders were controlled for (van Mourik et al., 2005, 2009; Albrecht et al., 2008b), but further improvements on how interference liability can be derived from performance data may clarify these findings (Lansbergen et al., 2007; Schwartz and Verhaeghen, 2008).

Thus, ADHD may be associated with a number of cognitive deficits, but these may not “causes” but rather consequences of the disorder, and may not provide causative therapy options: a recent meta-analysis suggests that cognitive trainings (e.g., on working memory) may improve performance and may ameliorate neuropsychological deficits found in ADHD, but direct effects on ADHD symptoms may be limited (Cortese et al., 2015).

#### Action Monitoring and Response Inhibition

Action monitoring as an important aspect of executive functioning comes into play when task demands raised response conflicts. There is a large body of evidence from electrophysiological studies elucidating some of the implicated mechanisms. For instance, if a task requires responding to a certain stimulus but to withhold the response to another one, the stimulus-locked event-related potentials (ERP) usually shows a fronto-central negativity peaking around 200–400 ms after onset of the stimulus which is larger for the Nogo than for the Go condition, particularly when the Nogo condition is rare. The same effect can be observed when the target is primed with either congruent or incongruent distractors. The so called N2 and the N2-enhancement were originally attributed to (response) inhibition (Kok, 1986, 1999; Falkenstein et al., 1999), but recent studies suggest that it reflects a more general action monitoring or cognitive control process that is also present if no response needs to be inhibited (Nieuwenhuis et al., 2003; Donkers and van Boxtel, 2004). Sources of N2 evoked by Go/Nogo- or Flanker-Tasks were found in medial frontal brain regions, namely the ACC (Van Veen and Carter, 2002; Nieuwenhuis et al., 2003; Bekker et al., 2005).

While several studies using Continous Performance Tests (CPT) or Go-Nogo-tasks in children did not find conflict-specific differences in N2 between ADHD and controls (Overtoom et al., 1998; Banaschewski et al., 2004; Fallgatter et al., 2004), some studies did, but variations were explained by comorbidity with other externalizing disorders (Lawrence et al., 2005; Wiersema et al., 2006) or appeared only with prolonged time-on-task (Yong-Liang et al., 2000). Thus, the detection of action monitoring deficits in ADHD may require tasks that are particularly demanding, e.g., that reveal a substantial number of performance errors, which is usually not realized in the CPT.

This may be achieved with the Flanker Task, requiring response to a central target flanked by either congruent or incongruent flanker stimuli (Eriksen and Eriksen, 1974) which was frequently used in ADHD research (Jonkman et al., 1999; Mullane et al., 2009). In a special variant of this task aimed maximizing the congruency effect, lower N2-enhancement and deficits during error processing in children and adults with ADHD, and moreover intermediate effects in first-degree relatives without a diagnosis of ADHD were found, indicating that action monitoring may be an important feature on the developmental pathway from genetical and environmental liability to ADHD (Albrecht et al., 2008a; McLoughlin et al., 2009).

Studies on brain activity more specifically related to response inhibition revealed mixed results, which may be explained by heterogeniety of the methods used. Studies with the Stop-Task, requiring a frequent and consequently predominant response which should be withold if a Stop-signal is presented, indicated that particularly the right inferior frontal gyrus is implicated in successful stopping of an ongoing response (Aron et al., 2003; Hughes et al., 2013). Several EEG and fMRI studies suggest impairments in Stop-Task performance and stop-signal related brain activity in ADHD (Brandeis et al., 1998; Pliszka et al., 2000; Albrecht et al., 2005; Rubia et al., 2008), but there are also some negative findings in treatment-naive children (Pliszka et al., 2006). Response inhibition problems in the Stop-Task may be significant in ADHD across the lifespan, and its specificity is particularly clear in adults with ADHD (Lijffijt et al., 2005).

Importantly, activity in the medial prefrontal cortex related to cognitive control (particularly the N2) and error processing (error negativity) may operate with theta (or maybe even lower delta) frequency (Yordanova et al., 2004; Cavanagh et al., 2012; Zavala et al., 2014).

##### Perspectives for NF

A number of recent studies with healthy adults indicate that NF training of frontal midline theta activity may improve attention and executive functions like working memory and cognitive flexibility (Wang and Hsieh, 2013; Enriquez-Geppert et al., 2014), and may lead to morphological changes in the cingulate cortex (Enriquez-Geppert et al., 2013). An application in ADHD may thus ameliorate cognitive deficits accordingly, but empirical evidence for improvement in executive functioning following NF in ADHD is weak (Vollebregt et al., 2014), and requires better controlled studies with sufficiently sized samples before definitive conclusions can be drawn.

A promising approach may be NF from dedicated brain regions that show diminished functional activity in ADHD. A recent study reported in this issue using near infrared spectroscopy (NIRS) NF of brain activity in the bilateral prefrontal cortex (implicated in executive functions and response inhibition) showed effectiveness in behavioral symptom ratings and executive functions, but may require fewer sessions than EEG or EMG NF (Marx et al., 2015). Activity in the ACC may be directly trained by tomographic NF (Bauer and Pllana, 2014). In a more recent study using tomographic NF from Theta/Beta or SCP activity localized in the ACC, Liechti et al. (2012) reported clinical improvement as well as resting EEG normalization in participants, but it remains open whether these improvements were (region-) specific for tomographic NF training (Liechti et al., 2012).

#### Preparation

Preparation for an upcoming event may be of great importance not only for specialists like flight controller, carefully watching their radar equipment for cues indicating critical situations that demand intervention. Almost half a century ago, it was found by Walter et al. that cues (predicting a consecutive imperative stimulus requiring a response) evoke a centrally negative SCP that terminates with the presentation of the next stimulus (contingent negative variation, CNV; Walter et al., 1964). Originally interpreted as “sensorimotor association and expectancy”, neuronal networks generating the CNV may be active if more general preparation for an upcoming event is required (Macar and Vidal, 2003).

Neurophysiological studies suggested that the CNV is generated in thalamo-cortical structures including the dorsal anterior cingulate cortex (ACC), frontal cortex, thalamus and midbrain dopaminergic nuclei (Gómez et al., 2003; Fan et al., 2007; Lütcke et al., 2008). Patients suffering from Parkinson’s disease that goes along with neuronal cell death in these nuclei showed specific reductions in Cue- (or warning stimulus) CNV amplitude (Pulvermüller et al., 1996; Ikeda et al., 1997; Gerschlager et al., 1999) as well as deficits in performance and slow wave activity during a temporal anticipation paradigm (Praamstra and Pope, 2007). This confirms the role of midbrain dopaminergic neurons in anticipation, time estimation or temporal memory (Suri and Schultz, 2001; Macar and Vidal, 2003).

Dopaminergic deficits may also explain anticipation and preparation problems in patients with ADHD, which showed reduced activation in brain regions implicated in CNV generation (Rubia et al., 1999; Smith et al., 2008). In line with these considerations, CNV is probably reduced in ADHD (van Leeuwen et al., 1998; Hennighausen et al., 2000; Perchet et al., 2001; Banaschewski et al., 2003a, 2008) and may represent a persistent deficit in patients with ADHD throughout life (McLoughlin et al., 2010; Doehnert et al., 2013). Moreover, diminished Cue-CNV may be familially-driven in children and adults with ADHD (McLoughlin et al., 2011; Albrecht et al., 2013) and may be related to polymorphisms of the dopamine receptor D4 gene (Albrecht et al., 2014). It is further subject to dopaminergic manipulations used for treatment of ADHD, as performance and CNV amplitude may be enhanced by methylphenidate (Linssen et al., 2011; Kratz et al., 2012).

##### Perspectives for NF

Many psychiatric or neurologic disorders are associated with preparation problems or related difficulties NF training of SCP may be a direct compensatory approach, as it relies on phasic modulation of SCPs and probably consequent cortical excitability (Rockstroh et al., 1984; Mayer et al., 2013; Gevensleben et al., 2014b).

Change in CNV-activity after SCP training is often replicated in NF-ADHD research (Gevensleben et al., 2012), but the relation to task performance appear rather complex and requires further investigation (Gevensleben et al., 2014a).

### Reward Processing

Deficient reward processing is a central aspect of several theories on ADHD. A model proposed by Sagvolden and colleagues claims that rewards have a shorter-term impact on learning and behavior in ADHD, e.g., characterized by a steeper gradient between the delay of a reinforcer and its effect on the probability that the reinforced action will be repeated (Sagvolden et al., 1998). Such a steeper delay of reinforcement gradient may be a consequence of lower tonic levels of dopaminergic activity in the mesolimbic system including the ventral tegmentum and the nucleus accumbens, while attention and response organization problems may originate from hypofunctioning of the mesocortical system also including the ventral tegmentum with projections to the prefrontal cortex (Sagvolden et al., 2005). Another model by Tripp and Wickens argues that phasic dopaminergic activity in the striatum related to cues indicating reinforcement may be impaired in ADHD (Tripp and Wickens, 2008).

A recent review by Plichta and Scheres summarizes consistent evidence from functional imaging studies on reward anticipation in ADHD: particularly the areas in the ventral striatum including nucleus caudatus, nucleus accumbens and the putamen show lower activation during reward anticipation in ADHD than controls, which may be rather related to hyperactive-impulsive symptom severity but perhaps not inattention (Plichta and Scheres, 2014).

#### 

##### Perspectives for NF

Immediate performance feedback may be beneficial for patients having problems with motivation or reinforcement anticipation. This would suggest that NF would be particularly applicable to such patients, but it may further modified to train brain activity associated with delayed reinforcement. As such, NF intervention may also help acquiring self-regulation skills useful for compensating motivational deficits and delay aversion in structured and potentially unattractive and boring situations.

### Resting State Brain Activity

Brain activity at rest, recorded when individuals are awake, relaxed and not engaged in any particular task, is characterized by complex oscillations that may reflect important features of arousal and attention that may change with development. Important aspects of resting state brain activity can be obtained using recordings of the EEG (Banaschewski and Brandeis, 2007; Rothenberger, 2009). The resting EEG of a time interval can be decomposed by means of a Fourier-Transformation in frequency and power. Cross-sectional developmental studies suggest that from childhood to adolescence and early adulthood a decrease in power of slow Delta (1.3–3.5 Hz) and Theta (3.5–7.5 Hz), but at the same time an increase in faster Alpha (7.5–12.5 Hz) and Beta (12.5 − ~25 Hz) activity emerges (Matousek and Petersen, 1973; John et al., 1982).

Earlier studies suggest that children with learning disabilities (Harmony et al., 1995), dyslexia (Klimesch et al., 2001) and ADHD (Bresnahan et al., 1999) may be characterized by lower power in the faster Alpha and Beta frequency bands, and in case of ADHD also potentially increased Theta activity (Barry et al., 2003). This view has been challenged by recent studies that did not replicate increased theta or theta/beta ratios in ADHD under resting conditions (Barry and Clarke, 2013; Liechti et al., 2013), albeit reduced relative beta power may be characteristic for a subgroup of children and adults with ADHD inattentive subtype (Buyck and Wiersema, 2014). A recent meta-analysis concludes that Theta/Beta-Ratio may not be a reliable diagnostic parameter in ADHD (Arns et al., 2013). However, there is some evidence that aberrances in EEG-frequency bands exist during task processing (El-Sayed et al., 2002). In a recent trial, Heinrich et al. found higher theta and alpha activity during an attentive state in children with ADHD, most pronounced in the upper theta/lower alpha range (5, 5–10, 5 Hz; Heinrich et al., 2014). Taken together, elevated power in lower frequency bands during rest may not be generally associated with ADHD, but there is some evidence that abnormalities of brain activity oscillations at least during task processing (in the “active brain”) might be part of the problem in children with ADHD.

Another view on resting state activity comes from a network perspective. MRI studies during rest (when participants were awake and rested quietly with eyes closed) revealed coherent activity fluctuations with low frequency (<0.1 Hz; Biswal et al., 1995) in a neuronal network including the medial prefrontal cortex, posterior cingulate cortex, precuneus and lateral parietal cortex (Gusnard et al., 2001a,b). This DMN activity is associated with a rather introspective and self-referential state (Gusnard et al., 2001a), which is attenuated during task performance when specific “task-positive” networks take over Fox et al., 2005; Fransson (2006). However, DMN activity may come back into play before performance errors or prolonged reaction times, possibly indicating attentional lapses (Weissman et al., 2006; Li et al., 2007; Eichele et al., 2008).

Misguided DMN activity may be important in several mental disorders (Broyd et al., 2009), and problems in ADHD may be particularly associated with attentional lapses due to DMN interference with activity in task-positive networks (Sonuga-Barke and Castellanos, 2007; Castellanos and Proal, 2012). Recent studies in adults with ADHD suggest lower anti-correlation between the posterior cingulate/precuneus (as an important part of the DMN) and the ACC often implicated in cognitive control and preparation (Castellanos et al., 2008; Uddin et al., 2008).

The association between DMN and electrical brain activity appears rather complex (Mantini et al., 2007) and may be unstable over time (Meyer et al., 2013). However, there are reports that very low frequency electrical brain activity (<1.5 Hz) may be altered in children and adults with ADHD, and particularly adults with higher ADHD symptom ratings show diminished deactivation in DMN regions during a flanker-task (Helps et al., 2010; Broyd et al., 2011).

#### 

##### Perspectives for NF

The theta/beta ratio during rest may not be generally impaired in patients with ADHD, but there is some evidence that problems may be present during task performance. Since NF targets the “active brain”, it may act as a potentially ameliorating intervention. Training on theta/beta ratio was successfully applied in a series of intervention studies in ADHD children, but the precise mode of action is still under investigation (Heinrich et al., 2007; Gevensleben et al., 2009). NF training targets different variables on the neurophysiological (enhancement of regulation capability of different EEG parameters), neuropsychological (executive functions), and the cognitive-behavioral (e.g., enhanced self-regulation by positive reinforcement of goal-directed behavior) level. Until now, it remains open whether regulation capability on the neurophysiological, the neuropsychological (executive functions), or on the cognitive-behavioral level—targeting an initial deficit or activating compensatory mechanisms—account for NF training effects (Gevensleben et al., 2014b). Most likely, NF outcome in ADHD treatment results from a combination of several of these variables.

Further venues of NF interventions may consider DMN interference by training the interplay between and connectivity within in the DMN and task-relevant networks.

## Heterogeniety in ADHD

ADHD is in many ways a heterogeneous disorder. This is reflected in the ADHD subtypes, overrepresentation of boys, and moreover in the fact that various comorbid conditions are not an exception, but the rule in patients with ADHD. For the presentation below, we consider comorbidities with a higher prevalence than the simple product of the prevalence of both disorders involved. As an example, the prevalence of oppositional defiant or conduct disorder (ODD/CD) in ADHD should be equal to the prevalence in the total population—in fact it is at least 20-times higher, and the reasons for this are still under debate. Research indicates that some comorbidities may in fact be a separate clinical entity (potentially like ADHD + ODD/CD, as discussed below), whilst others may in many ways an addition of difficulties present in either disorder (e.g., like ADHD + Tic). In any case, heterogeneity in ADHD may further complicate treatment.

### Clinical Heterogeneity—Hyperactive/Impulsive and Inattentive Subtypes

The Diagnostic and Statistical Manual distinguishes Hyperactive/Impulsive (ADHD-H) and Inattention (ADHD-I) symptom clusters in the diagnosis of ADHD (APA, 2013), but it remains controversial whether these form separate clinical entities. On the one hand, developmental studies suggest that children initially diagnosed with ADHD-H may shift to Combined Type (ADHD-C) as attention demands increase in school, whilst diagnoses of attention problems alone may remain stable and form a separate clinical entity (Lahey et al., 2005). Compared to patients with ADHD combined type, children with ADHD-I may be characterized by rather passive social interaction and more associated internalizing problems (Maedgen and Carlson, 2000). In the resting EEG, children and adults with ADHD-I may show lower power in the beta-band and increased theta/beta ratio (Buyck and Wiersema, 2014), which may have significance for compensatory NF intervention.

However, on the neuropsychological level children with ADHD-I show similar performance problems as ADHD-C in a wide range of demands (Nigg et al., 2002; Baeyens et al., 2006), albeit there is some evidence that ADHD-I may show particular preparation problems (Adams et al., 2008).

#### 

##### Perspectives for NF

Empirical evidence suggests that NF unfolds an impact on all three symptom clusters of ADHD (inattention, impulsivity, hyperactivity). In a large multicenter randomized controlled trial of a combination of theta-beta/SCP NF training for children with ADHD, we found comparable effects for symptoms of inattention as well as hyperactivity/impulsivity (Gevensleben et al., 2014b). No differences in efficacy concerning subtypes of ADHD were obtained. This result is supported by meta-analytic data, obtaining large effect sizes for inattention and medium to large effect sizes for hyperactivity/impulsivity symptom ratings (Arns et al., 2014). Latest evidence from a trial encompassing children with comorbidity of Tourette-disorder and ADHD suggested that specificity of outcome of NF training concerning patterns of inattention, hyperactivity and or impulsivity may rely on transfer tasks (homework) in the course of the training (Gevensleben et al., 2014b), which should be considered in the treatment of ADHD subtypes.

### Similarities and Differences Between Boys and Girls with ADHD

Although overrepresentation of boys in ADHD is at least 3 to 1 in the population and much higher among clinical referrals (Tannock, 1998; APA, 2013), studies explicitly addressing the role of sex on cognitive parameters are rare. In an earlier meta-analysis, Gershon concluded that girls suffering from ADHD were lower-rated on ADHD symptoms and externalizing problems, but they were more impaired than boys on internalizing symptoms. Furthermore, females showed lower “crystallized” cognitive functioning as measured by full scale and verbal IQ, but “fluid” performance IQ did not differ between sexes. Regarding executive functions, girls with ADHD show in many ways similar impairments as boys (Gershon, 2002).

That has been demonstrated with several neuropsychological tests including the Stroop- (deHaas, 1986; Rucklidge and Tannock, 2002) and Wisconsin Card Sorting Test (Houghton et al., 1999; Seidman et al., 2005) as well as with various versions of the CPT (Breen, 1989; Schuerholz et al., 1998; Sharp et al., 1999; Yang et al., 2004; Seidman et al., 2006). An exception may be impulsivity, or consequent problems with response inhibition: a recent meta-analysis on CPT performance by Hasson and Fine indicated that girls may generally show less commission errors than boys, and beyond that case-control differences on impulsivity errors were also lower in girls (Hasson and Fine, 2012).

Studies on brain activity during preparation and response control yielded mixed results. Regarding cognitive control, Liotti et al. (2007) found no sex differences in Stop-Task performance and N2 amplitude (Liotti et al., 2007). This is in line with a more recent study with the Flanker-Task, detecting independently of sex also problems with N2-enhancement and error processing in nonaffected siblings of patients with ADHD (girls were outnumbered in our ADHD sample, not allowing a direct comparison in patients), albeit girls showed a generally more accurate response style and larger error positivity probably associated with affective error processing (Albrecht et al., 2010). In the CPT on familiarity, girls made less commission errors (particularly in a more demanding CPT with additional incompatible flanker stimuli) at the expense of slower response speed, and they also showed larger Cue-P3, but similar Cue-CNV (Albrecht et al. in preparation).

Taken together, girls with ADHD or nonaffected siblings may show a rather accurate response style and fewer problems with impulsivity, but they may share many problems with executive functions detected in studies mostly on boys. It remains an open question whether impulsivity explains the overrepresentation of boys among patients with ADHD as it may lead to more severe and probably clinically relevant psychosocial impairments.

#### 

##### Perspectives for NF

Since girls with ADHD may show similar impairments as boys, although they may be less impulsive, the current literature suggests that NF interventions need no sex-specific adaptations, but more research is needed before definitive recommendations can be given.

### ADHD and Conduct Disorder

Children with conduct problems display a repetitive and persistent pattern of oppositional or dissocial behavior, aggression, or deliquency for more than 6 months that goes beyond childlike mischief or typical problems during puberty. The DSM distinguishes ODD, characterized by “negativistic, hostile, and defiant behavior” from CD including aggression, violation of “the basic rights of others”, and delinquency (APA, 2013). Since ODD and CD often occur interrelated and the former may antecede the latter in child development, both are often considered together as ODD/CD (Loeber et al., 2000).

With a prevalence of approximately 2% in children and adolescents, it is one of the more frequent child psychiatric diagnoses. However, among patients with ADHD, ODD/CD is with a comorbidity rate of 40–70% much more frequent, and the reasons for this are still under investigation (Newcorn and Halperin, 2005). It was found that ADHD may be a predictor for ODD, and that ODD may predict CD (Burke et al., 2005).

ODD/CD is associated with a number of neurobiological abnormalities. At first, children with ODD/CD may show impaired stress reactivity, which is mediated by activity in the hypothalamic-pituitary-adrenal (HPA) axis and expressed in the release of cortisol, which was moderately inverse associated with aggressive symptoms (van Goozen et al., 2007). This may result in low sensitivity to punishment that may in turn hamper learning from the consequences of inappropriate behavior (Matthys et al., 2013). Further, activity of the autonomic nervous system (ANS), via the interplay of its sympathetic and parasympathetic branches responsible for the regulation of arousal and energy generation, may be lower (as indicated by lower heart rate and skin conductance reactivity) in aggressive or antisocial individuals (Lorber, 2004), suggesting underarousal and consequently “fearlessness” (Raine, 2002) or risk taking and “sensation seeking” (Zuckerman, 1994). Moreover, there are also studies linking lower serotoninergic activity (5-HT) and monoamine-oxydases (MAO) to aggression, and selective serotonine reuptake inhibitors are frequently used for reducing aggression in patients (Carrillo et al., 2009).

In a series of neuroimaging experiments, Rubia et al. showed that children with ADHD may be characterized by “cool” cognitive deficits like inhibition, attention and timing related to abnormal activity in inferior frontal, striatal and parietotemporal brain regions, whereas ODD/CD was associated with “hot” deficits in the regulation of motivation and affect (related to emotional impulsivity) resulting from dysfunctions in the paralimbic system including orbito-frontal and superior-temporal areas and the ACC as well as the limbic system (Rubia et al., 2008, 2009a,b; Rubia, 2011). Particularly the latter may lead to a lack of self-control in emotional situations Matthys et al. (2013).

Besides these differences between ADHD and ODD/CD, there is an ongoing debate, whether children with comorbid ADHD + ODD/CD may form a separate clinical entity as diagnosed in the ICD-10 as “F90.1 Hypercinetic Conduct Disorder” (WHO, 1993), or whether the comorbidity of ADHD and ODD/CD may be considered separately as in the DSM-V (APA, 2013).

In earlier electrophysiological studies, Banaschewski et al. studied children with ADHD, ODD/CD, comorbid ADHD + ODD/CD and controls in a 2*2-factorial design assessing additive and non-additive effects of both disorders. During CPT performance, children with ADHD showed slower and more variable response speed, whilst children with comorbid ADHD + ODD/CD committed more dyscontrol errors. On the level of brain electrical activity, children with pure ADHD were characterized by lower Cue-CNV suggesting preparation problems. Both children with pure ADHD and ODD/CD but not comorbid ADHD + ODD/CD displayed diminished attentional orientation as indicated by impaired P3a to Cues and uncued targets (Banaschewski et al., 2003a). Children with comorbid ADHD + ODD/CD displayed diminished Nogo-P3 related to motor response control (Banaschewski et al., 2004). In the Stop-Task, response inhibition deficits in performance and associated brain activity of both children with pure ADHD and ODD/CD reached significance, whilst the yougsters with comorbid ADHD + ODD/CD did again show overall less severe problems (Albrecht et al., 2005). These findings are supported in a neuropsychological study by Luman et al showing that children with ADHD + ODD/CD were located in between children with ADHD and controls regarding response inhibition speed, timing performance and the impact of incentives or penalty on timing performance (Luman et al., 2009).

Taken together, children with ADHD and ODD/CD may have a broad deficit in performance and brain activity associated with several executive functions. Comorbid ADHD + ODD/CD may show elevated “hot” cognitive deficits in motivation, affect regulation and impulsivity (and accordingly response control problems), whilst problems with “cool” attentional orienting and response preparation may be less severe than expected from an additive model of impairments found in both pure ADHD and ODD/CD.

#### 

##### Perspectives for NF

There is evidence that children with comorbid ADHD + ODD/CD may show particularly problems with self-regulation, whilst cognitive deficits may be less severe than expected from typical findings from ADHD and ODD/CD, suggesting that NF interventions may use these cognitive resources and may dwell on the enhancement or compensation of diminished self-regulation skills accordingly. Since ADHD may precede ODD/CD in child development, early interventions may be particularly promising. Moreover, combined NF SCP and theta/beta training yielded, besides particular reductions of teacher and parent-rated ADHD symptoms, also effects on parent-rated oppositional behavior and conduct problems when compared to a standardized computer attention training (Gevensleben et al., 2009), indicating that NF may also be beneficial in treating ODD/CD.

### ADHD and Tic Disorder

Tic disorders (TD) are characterized by involuntary, sudden, short, repetitive and non-rhythmic fragments of usual movements and/or vocal expressions. Tics are ranging from mild (e.g., eye blinking or sniffing) to severe (e.g., strong head or body jerking, shouting) intensity and simple (e.g., shoulder shrugging, grunting) to complex (e.g., turning, vocalizing complex words or sentences) extent that do not fulfil any subjective purpose (Leckman, 2002). TD are considered as “Neurodevelopmental Disorders” in the DSM-V (APA, 2013); more details about clinical assessment and treatment options may be found in the European Guidelines on TD (Cath et al., 2011; Muller-Vahl et al., 2011; Roessner et al., 2011; Verdellen et al., 2011).

Tics do often occur in bouts and are particularly present during stress or in positive or negative emotional situations. Particularly patients older than 10 years may realize sensory-motor phenomena (e.g., an urge to execute a tic) before and/or after tic, and are often able to suppress their tics for a limited period of time (Banaschewski et al., 2003b). Urges and uneasy sensory-motor feelings may be a part of the impairment (Swain et al., 2007).

The situation of patients with Tics is further complicated by frequent comorbidities with ADHD, anxiety, obsessive-compulsive disorder or mood disorder. ADHD + TD may be present in about half of children with TD, particularly in patients with Tourette’s syndrome.

Assessment of the psychopathological profile revealed particularly mood, thought, attention and social problems as well as somatic complaints in children with TD, which do partly overlap with difficulties found in ADHD. Importantly, effects were (with the exception of somatic complaints) additive in ADHD and TD (Roessner et al., 2007).

TD is probably associated with disturbances in cortico-striato-thalamo-cortical neuronal networks which may be partly compensated by increased prefrontal activity instrumental in tic suppression (Leckman et al., 2006; Swain et al., 2007; Wang et al., 2011). Studies on structural imaging via voxel-based morphometry (VBM) or diffusion tensor imaging showed abnormalities in a number of brain regions including the basal ganglia, putamen, thalamus, corpus callosum and in the prefrontal cortex, which may reflect pathological as well as compensatory alterations (Peterson et al., 2003; Plessen et al., 2004; Jackson et al., 2011; Liu et al., 2013; Müller-Vahl et al., 2014). However, a recent VBM study with younger medication naïve boys with Tourette’s syndrome without comorbid conditions did not replicate abnormalities in gray or White matter, (Roessner et al., 2009). Thus, it remains open whether the above mentioned structural abnormalities may be a consequence of long-term tic suppression or comorbidities in Tic disorders.

A recent imaging study in adults generally replicated an earlier study of Bohlhalter by further elucidating the progression of brain activity in the cortico-striato-thalamo-cortical circuit preceding a tic and additionally suggesting the role of DMN activity in tic generation (Bohlhalter et al., 2006; Neuner et al., 2014). Peterson found increased activity during tic suppression in the prefrontal cortex and basal ganglia, which were inversely related to tic severity in everyday life (Peterson et al., 1998).

Moreover, studies on excitability of the motor system using transcranial magnetic stimulation indicated reduced motor inhibition in individuals with TD (Ziemann et al., 1997), which improves with development (Moll et al., 2006). Reduced inhibition within the motor circuit was also seen in ADHD, and shows again additive effects in individuals with comorbid ADHD + TD (Moll et al., 2001; Orth and Rothwell, 2009; for a review see Orth, 2009).

Studies on higher order cognitive functions in TD suggest that patients may not show general impairments, but may at most be affected as a consequence of their tics or potential compensatory mechanisms; studies addressing the co-existence of TD + ADHD often found deficits explained by ADHD following an additive model (Schuerholz et al., 1998; Roessner et al., 2008; Greimel et al., 2011). However, this may not hold for electrophysiological parameters of preparation and self-regulation after decision making, as children with ADHD + Tic may be more similar to children with pure Tic disorder, following a sub-additive model (Yordanova et al., 1996, 1997). Diminished preparatory slow-wave CNV in children with Tics was furthermore associated with tic severity, suggesting a possible functional link with tic suppression (Siniatchkin and Kuppe, 2011).

Functional imaging studies particularly in adults with TD yielded mixed results, and the interpretation of functional data in TD may be complicated by the interplay of both activity reflecting pathophysiological mechanisms and potential compensatory activity required during tic control (Gerard and Peterson, 2003; Vloet et al., 2006). This is supported by a cross-sectional developmental study in children and adults with the Stroop-task, showing normal performance during interference control, but elevated fronto-striatal activity and deviant development of activity in prefrontal and posterior cingulate areas in patients with Tic disorder compared to healthy controls (Marsh et al., 2007).

#### 

##### Perspectives for NF

In sum, ADHD + TD is probably not a separate clinical entity as it is the case for ADHD + ODD/CD. Individuals with ADHD + TD may share difficulties associated with both disorders often following an additive model, which may however be a special challenge to treat. SCP-NF may be used to ameliorate diminished slow-wave activity during preparation, which may be a common psychopathological impairment in both ADHD and Tic disorders. Typical interventions used in the treatment of TD that rely on intact cognitive control mechanisms or executive functions such as habit reversal may be less effective when ADHD is associated.

Nevertheless, NF may be a promising component of a multi-modal therapy, as it combines training of potentially problematic brain activity with direct feedback and reward.

In previous intervention studies, Theta-SMR-protocols have been applied in Tic-/Tourette disorder, from a traditionally point of view aiming at inhibiting over-activity in senso-motor cortical regions (Tansey, 1986). There is some evidence for positive effects of NF in subjects with Tic-/Tourette disorder from several single-case trials (see Rothenberger and Gevensleben, 2013 for a short overview). Further promising results evolved from a pilot study comparing the impact of sensory motor rhythm (SMR; 12–15 Hz) vs. SCP-NF training on Tic-/Tourette symptoms (Gevensleben et al., 2014b). Under both conditions Tic-severity was reduced by about 25% after 24 units of NF. Interestingly, in those patients who additionally suffered from ADHD (about 50% of the sample) ADHD symptoms are reduced significantly only after SCP training (not after SMR), indicating a potentially specific effect of SCP training on ADHD (at least in patients suffering from comorbid ADHD + TD).

## Conclusion

ADHD as a neurodevelopmental disorder is associated with pathophysiological problems during cognitive demands, reward processing and during rest. It is further complicated by a number of heterogeneities regarding clinical characteristics, sex differences, and frequent comorbid disorders. The current manuscript introduced associated pathophysiological characteristics and discussed their potential relevance for NF intervention in ADHD.

It is argued that cognitive deficits during preparation for an upcoming event and response inhibition problems associated with deficient activity in the prefrontal cortex and in the ACC, as well as impaired resting-state brain activity may be ameliorated by respective NF trainings. Tomographic NF interventions using high density EEG recordings, fMRI or NIRS targeting specific brain areas may allow more direct training of brain activity impaired in ADHD. Motivational problems during reward processing may rather be compensated by the acquisition of self-regulation skills.

NF interventions may be used for hyperactive/impulsive and inattentive subtypes, and may not require sex-specific adaptations. It may in general be useful for children with comorbid ADHD and conduct disorders characterized in many ways by sub-additive cognitive deficits of both pure disorders but pronounced self-regulation deficits, as well as for ADHD and associated Tic disorder characterized largely by additive impairments.

Taken together, NF intervention in ADHD may be applied in order to ameliorate specific deficits, and/or to acquire self-regulation skills to use them for the compensation of difficulties in other domains.

## Conflict of Interest Statement

The authors declare that the research was conducted in the absence of any commercial or financial relationships that could be construed as a potential conflict of interest.

## References

[B1] Adams Z. W. Derefinko K. J. Milich R. Fillmore M. T. (2008). Inhibitory functioning across ADHD subtypes: recent findings, clinical implications and future directions. Dev. Disabil. Res. Rev. 14, 268–275. 10.1002/ddrr.37 19072751 PMC4131681

[B2] Albrecht B. Banaschewski T. Brandeis D. Heinrich H. Rothenberger A. (2005). Response inhibition deficits in externalizing child psychiatric disorders: an ERP-study with the stop-task. Behav. Brain Funct. 1:22. 10.1186/1744-9081-1-22 16336676 PMC1343568

[B3] Albrecht B. Brandeis D. Uebel H. Heinrich H. Heise A. Hasselhorn M.. (2010). Action monitoring in children with or without a family history of ADHD–effects of gender on an endophenotype parameter. Neuropsychologia 48, 1171–1177. 10.1016/j.neuropsychologia.2009.12.018 20026087 PMC2878640

[B4] Albrecht B. Brandeis D. Uebel H. Heinrich H. Mueller U. C. Hasselhorn M.. (2008a). Action monitoring in boys with attention-deficit/hyperactivity disorder, their nonaffected siblings and normal control subjects: evidence for an endophenotype. Biol. Psychiatry 64, 615–625. 10.1016/j.biopsych.2007.12.016 18339358 PMC2580803

[B5] Albrecht B. Brandeis D. Uebel H. Valko L. Heinrich H. Drechsler R.. (2013). Familiality of neural preparation and response control in childhood attention deficit-hyperactivity disorder. Psychol. Med. 43, 1997–2011. 10.1017/s003329171200270x 23200032

[B6] Albrecht B. Brandeis D. Uebel-Von Sandersleben H. Valko L. Heinrich H. Xu X.. (2014). Genetics of preparation and response control in ADHD: the role of DRD4 and DAT1. J. Child Psychol. Psychiatry 55, 914–923. 10.1111/jcpp.12212 24521003

[B7] Albrecht B. Rothenberger A. Sergeant J. Tannock R. Uebel H. Banaschewski T. (2008b). Interference control in attention-deficit/hyperactivity disorder: differential stroop effects for colour-naming versus counting. J. Neural Transm. 115, 241–247. 10.1007/s00702-007-0818-1 17896071

[B8] APA (2013). Diagnostic and Statistical Manual of Mental Disorders, Fifth Edition, DSM-5. Arlington, VA: American Psychiatric Association.

[B9] Arns M. Strehl U. (2013). Evidence for efficacy of NF in ADHD? Am. J. Psychiatry 170, 799–800. 10.1176/appi.ajp.2013.13020208 23820843

[B10] Arns M. Conners C. K. Kraemer H. C. (2013). A decade of EEG theta/beta ratio research in ADHD: a meta-analysis. J. Atten. Disord. 17, 374–383. 10.1177/1087054712460087 23086616

[B11] Arns M. Heinrich H. Strehl U. (2014). Evaluation of NF in ADHD: the long and winding road. Biol. Psychol. 95, 108–115. 10.1016/j.biopsycho.2013.11.013 24321363

[B12] Aron A. R. Fletcher P. C. Bullmore E. T. Sahakian B. J. Robbins T. W. (2003). Stop-signal inhibition disrupted by damage to right inferior frontal gyrus in humans. Nat. Neurosci. 6, 115–116. 10.1038/nn1203-1329a 12536210

[B13] Baddeley A. Della Sala S. (1996). Working memory and executive control. Philos. Trans. R Soc. Lond. B Biol. Sci. 351, 1397–1403; discussion 1403–1394. 10.1098/rstb.1996.0123 8941951

[B14] Baeyens D. Roeyers H. Walle J. V. (2006). Subtypes of attention-deficit/hyperactivity disorder (ADHD): distinct or related disorders across measurement levels? Child Psychiatry Hum. Dev. 36, 403–417. 10.1007/s10578-006-0011-z 16755403

[B15] Banaschewski T. Brandeis D. (2007). Annotation: what electrical brain activity tells us about brain function that other techniques cannot tell us - a child psychiatric perspective. J. Child Psychol. Psychiatry 48, 415–435. 10.1111/j.1469-7610.2006.01681.x 17501723

[B16] Banaschewski T. Brandeis D. Heinrich H. Albrecht B. Brunner E. Rothenberger A. (2003a). Association of ADHD and conduct disorder–brain electrical evidence for the existence of a distinct subtype. J. Child Psychol. Psychiatry 44, 356–376. 10.1111/1469-7610.00127 12635966

[B17] Banaschewski T. Brandeis D. Heinrich H. Albrecht B. Brunner E. Rothenberger A. (2004). Questioning inhibitory control as the specific deficit of ADHD–evidence from brain electrical activity. J. Neural Transm. 111, 841–864. 10.1007/s00702-003-0040-8 15206002

[B18] Banaschewski T. Coghill D. Santosh P. Zuddas A. Asherson P. Buitelaar J.. (2006). Long-acting medications for the hyperkinetic disorders. A systematic review and European treatment guideline. Eur. Child Adolesc. Psychiatry 15, 476–495. 10.1007/s00787-006-0549-0 16680409

[B19] Banaschewski T. Woerner W. Rothenberger A. (2003b). Premonitory sensory phenomena and suppressibility of tics in Tourette syndrome: developmental aspects in children and adolescents. Dev. Med. Child Neurol. 45, 700–703. 10.1111/j.1469-8749.2003.tb00873.x 14515942

[B20] Banaschewski T. Yordanova J. Kolev V. Heinrich H. Albrecht B. Rothenberger A. (2008). Stimulus context and motor preparation in attention-deficit/hyperactivity disorder. Biol. Psychol. 77, 53–62. 10.1016/j.biopsycho.2007.09.003 17964058

[B21] Barkley R. A. (1997). Behavioral inhibition, sustained attention and executive functions: constructing a unifying theory of ADHD. Psychol. Bull. 121, 65–94. 10.1037/0033-2909.121.1.65 9000892

[B22] Barkley R. A. Grodzinsky G. DuPaul G. J. (1992). Frontal lobe functions in attention deficit disorder with and without hyperactivity: a review and research report. J. Abnorm. Child Psychol. 20, 163–188. 10.1007/bf00916547 1593025

[B23] Barry R. J. Clarke A. R. (2013). Resting state brain oscillations and symptom profiles in attention deficit/hyperactivity disorder. Suppl. Clin. Neurophysiol. 62, 275–287. 10.1016/b978-0-7020-5307-8.00017-x 24053045

[B24] Barry R. J. Clarke A. R. Johnstone S. J. (2003). A review of electrophysiology in attention-deficit/hyperactivity disorder: I. Qualitative and quantitative electroencephalography. Clin. Neurophysiol. 114, 171–183. 10.1016/s1388-2457(02)00362-0 12559224

[B25] Bauer H. Pllana A. (2014). EEG-based local brain activity feedback training-tomographic NF. Front. Hum. Neurosci. 8:1005. 10.3389/fnhum.2014.01005 25566027 PMC4264468

[B26] Bekker E. M. Kenemans J. L. Verbaten M. N. (2005). Source analysis of the N2 in a cued Go/NoGo task. Brain Res. Cogn. Brain Res. 22, 221–231. 10.1016/j.cogbrainres.2004.08.011 15653295

[B27] Biederman J. (2005). Attention-deficit/hyperactivity disorder: a selective overview. Biol. Psychiatry 57, 1215–1220. 10.1016/j.biopsych.2004.10.020 15949990

[B28] Biswal B. Yetkin F. Z. Haughton V. M. Hyde J. S. (1995). Functional connectivity in the motor cortex of resting human brain using echo-planar MRI. Magn. Reson. Med. 34, 537–541. 10.1002/mrm.1910340409 8524021

[B29] Bohlhalter S. Goldfine A. Matteson S. Garraux G. Hanakawa T. Kansaku K.. (2006). Neural correlates of tic generation in Tourette syndrome: an event-related functional MRI study. Brain 129, 2029–2037. 10.1093/brain/awl050 16520330

[B30] Brandeis D. Van Leeuwen T. H. Rubia K. Vitacco D. Steger J. Pascual-Marqui R. D.. (1998). Neuroelectric mapping reveals precursor of stop failures in children with attention deficits. Behav. Brain Res. 94, 111–125. 10.1016/s0166-4328(97)00174-5 9708844

[B31] Breen M. J. (1989). Cognitive and behavioral differences in AdHD boys and girls. J. Child Psychol. Psychiatry 30, 711–716. 10.1111/j.1469-7610.1989.tb00783.x 2793958

[B32] Bresnahan S. M. Anderson J. W. Barry R. J. (1999). Age-related changes in quantitative EEG in attention-deficit/hyperactivity disorder. Biol. Psychiatry 46, 1690–1697. 10.1016/s0006-3223(99)00042-6 10624551

[B33] Broyd S. J. Demanuele C. Debener S. Helps S. K. James C. J. Sonuga-Barke E. J. (2009). Default-mode brain dysfunction in mental disorders: a systematic review. Neurosci. Biobehav. Rev. 33, 279–296. 10.1016/j.neubiorev.2008.09.002 18824195

[B34] Broyd S. J. Helps S. K. Sonuga-Barke E. J. (2011). Attention-induced deactivations in very low frequency EEG oscillations: differential localisation according to ADHD symptom status. PLoS One 6:e17325. 10.1371/journal.pone.0017325 21408092 PMC3050980

[B35] Burke J. D. Loeber R. Lahey B. B. Rathouz P. J. (2005). Developmental transitions among affective and behavioral disorders in adolescent boys. J. Child Psychol. Psychiatry 46, 1200–1210. 10.1111/j.1469-7610.2005.00422.x 16238667

[B36] Buyck I. Wiersema J. R. (2014). Resting electroencephalogram in attention deficit hyperactivity disorder: developmental course and diagnostic value. Psychiatry Res. 216, 391–397. 10.1016/j.psychres.2013.12.055 24656956

[B37] Carrillo M. Ricci L. A. Coppersmith G. A. Melloni R. H. Jr. (2009). The effect of increased serotonergic neurotransmission on aggression: a critical meta-analytical review of preclinical studies. Psychopharmacology (Berl) 205, 349–368. 10.1007/s00213-009-1543-2 19404614

[B38] Castellanos F. X. Proal E. (2012). Large-scale brain systems in ADHD: beyond the prefrontal-striatal model. Trends Cogn. Sci. 16, 17–26. 10.1016/j.tics.2011.11.007 22169776 PMC3272832

[B39] Castellanos F. X. Margulies D. S. Kelly C. Uddin L. Q. Ghaffari M. Kirsch A.. (2008). Cingulate-precuneus interactions: a new locus of dysfunction in adult attention-deficit/hyperactivity disorder. Biol. Psychiatry 63, 332–337. 10.1016/j.biopsych.2007.06.025 17888409 PMC2745053

[B40] Cath D. C. Hedderly T. Ludolph A. G. Stern J. S. Murphy T. Hartmann A.. (2011). European clinical guidelines for Tourette syndrome and other tic disorders. Part I: assessment. Eur. Child Adolesc. Psychiatry 20, 155–171. 10.1007/s00787-011-0164-6 21445723 PMC3065640

[B41] Cavanagh J. F. Zambrano-Vazquez L. Allen J. J. (2012). Theta lingua franca: a common mid-frontal substrate for action monitoring processes. Psychophysiology 49, 220–238. 10.1111/j.1469-8986.2011.01293.x 22091878 PMC3262926

[B42] Cortese S. Ferrin M. Brandeis D. Buitelaar J. Daley D. Dittmann R. W.. (2015). Cognitive training for attention-deficit/hyperactivity disorder: meta-analysis of clinical and neuropsychological outcomes from randomized controlled trials. J. Am. Acad. Child Adolesc. Psychiatry 54, 164–174. 10.1016/j.jaac.2014.12.010 25721181 PMC4382075

[B43] Daley D. van der Oord S. Ferrin M. Danckaerts M. Doepfner M. Cortese S.. (2014). Behavioral interventions in attention-deficit/hyperactivity disorder: a meta-analysis of randomized controlled trials across multiple outcome domains. J. Am. Acad. Child Adolesc. Psychiatry 53, 835–847. 10.1016/j.jaac.2014.05.013 25062591

[B44] deHaas P. A. (1986). Attention styles and peer relationships of hyperactive and normal boys and girls. J. Abnorm. Child Psychol. 14, 457–467. 10.1007/bf00915438 3760350

[B45] Diamond A. (2013). Executive functions. Annu. Rev. Psychol. 64, 135–168. 10.1146/annurev-psych-113011-143750 23020641 PMC4084861

[B46] Doehnert M. Brandeis D. Schneider G. Drechsler R. Steinhausen H. C. (2013). A neurophysiological marker of impaired preparation in an 11-year follow-up study of attention-deficit/hyperactivity disorder (ADHD). J. Child Psychol. Psychiatry 54, 260–270. 10.1111/j.1469-7610.2012.02572.x 22788246

[B47] Donkers F. C. van Boxtel G. J. (2004). The N2 in go/no-go tasks reflects conflict monitoring not response inhibition. Brain Cogn. 56, 165–176. 10.1016/j.bandc.2004.04.005 15518933

[B48] Eichele T. Debener S. Calhoun V. D. Specht K. Engel A. K. Hugdahl K.. (2008). Prediction of human errors by maladaptive changes in event-related brain networks. Proc. Natl. Acad. Sci. U S A 105, 6173–6178. 10.1073/pnas.0708965105 18427123 PMC2329680

[B49] El-Sayed E. Larsson J. O. Persson H. E. Rydelius P. A. (2002). Altered cortical activity in children with attention-deficit/hyperactivity disorder during attentional load task. J. Am. Acad. Child Adolesc. Psychiatry 41, 811–819. 10.1097/00004583-200207000-00013 12108806

[B50] Enriquez-Geppert S. Huster R. J. Figge C. Herrmann C. S. (2014). Self-regulation of frontal-midline theta facilitates memory updating and mental set shifting. Front. Behav. Neurosci. 8:420. 10.3389/fnbeh.2014.00420 25538585 PMC4257088

[B51] Enriquez-Geppert S. Huster R. J. Scharfenort R. Mokom Z. N. Vosskuhl J. Figge C.. (2013). The morphology of midcingulate cortex predicts frontal-midline theta NF success. Front. Hum. Neurosci. 7:453. 10.3389/fnhum.2013.00453 23950741 PMC3739027

[B52] Eriksen B. A. Eriksen C. W. (1974). Effects of noise letters upon the identification of a target letter in a nonsearch task. Percept. Psychophys. 16, 143–149. 10.3758/bf03203267

[B53] Falkenstein M. Hoormann J. Hohnsbein J. (1999). ERP components in Go/Nogo tasks and their relation to inhibition. Acta Psychol. (Amst) 101, 267–291. 10.1016/s0001-6918(99)00008-6 10344188

[B54] Fallgatter A. J. Ehlis A. C. Seifert J. Strik W. K. Scheuerpflug P. Zillessen K. E.. (2004). Altered response control and anterior cingulate function in attention-deficit/hyperactivity disorder boys. Clin. Neurophysiol. 115, 973–981. 10.1016/j.clinph.2003.11.036 15003781

[B55] Fan J. Kolster R. Ghajar J. Suh M. Knight R. T. Sarkar R.. (2007). Response anticipation and response conflict: an event-related potential and functional magnetic resonance imaging study. J. Neurosci. 27, 2272–2282. 10.1523/jneurosci.3470-06.2007 17329424 PMC6673473

[B56] Fayyad J. De Graaf R. Kessler R. Alonso J. Angermeyer M. Demyttenaere K.. (2007). Cross-national prevalence and correlates of adult attention-deficit hyperactivity disorder. Br. J. Psychiatry 190, 402–409. 10.1192/bjp.bp.106.034389 17470954

[B57] Fox M. D. Snyder A. Z. Vincent J. L. Corbetta M. Van Essen D. C. Raichle M. E. (2005). The human brain is intrinsically organized into dynamic, anticorrelated functional networks. Proc. Natl. Acad. Sci. U S A 102, 9673–9678. 10.1073/pnas.0504136102 15976020 PMC1157105

[B58] Fransson P. (2006). How default is the default mode of brain function? Further evidence from intrinsic BOLD signal fluctuations. Neuropsychologia 44, 2836–2845. 10.1016/j.neuropsychologia.2006.06.017 16879844

[B59] Gerard E. Peterson B. S. (2003). Developmental processes and brain imaging studies in Tourette syndrome. J. Psychosom. Res. 55, 13–22. 10.1016/s0022-3999(02)00581-0 12842227

[B60] Gerschlager W. Alesch F. Cunnington R. Deecke L. Dirnberger G. Endl W.. (1999). Bilateral subthalamic nucleus stimulation improves frontal cortex function in Parkinson’s disease. An electrophysiological study of the contingent negative variation. Brain 122, 2365–2373. 10.1093/brain/122.12.2365 10581229

[B61] Gershon J. (2002). A meta-analytic review of gender differences in ADHD. J. Atten. Disord. 5, 143–154. 10.1177/108705470200500302 11911007

[B62] Gevensleben H. Albrecht B. Lütcke H. Auer T. Dewiputri W. I. Schweizer R.. (2014a). NF of slow cortical potentials: neural mechanisms and feasibility of a placebo-controlled design in healthy adults. Front. Hum. Neurosci. 8:990. 10.3389/fnhum.2014.00990 25566020 PMC4263073

[B63] Gevensleben H. Holl B. Albrecht B. Vogel C. Schlamp D. Kratz O.. (2009). Is NF an efficacious treatment for ADHD? A randomised controlled clinical trial. J. Child Psychol. Psychiatry 50, 780–789. 10.1111/j.1469-7610.2008.02033.x 19207632

[B64] Gevensleben H. Kleemeyer M. Rothenberger L. G. Studer P. Flaig-Röhr A. Moll G. H.. (2014b). NF in ADHD: further pieces of the puzzle. Brain Topogr. 27, 20–32. 10.1007/s10548-013-0285-y 23563906

[B65] Gevensleben H. Moll G. H. Rothenberger A. Heinrich H. (2014c). NF in attention-deficit/hyperactivity disorder - different models, different ways of application. Front. Hum. Neurosci. 8:846. 10.3389/fnhum.2014.00846 25374528 PMC4204462

[B66] Gevensleben H. Rothenberger A. Moll G. H. Heinrich H. (2012). NF in children with ADHD: validation and challenges. Expert Rev. Neurother. 12, 447–460. 10.1586/ern.12.22 22449216

[B67] Gómez C. M. Marco J. Grau C. (2003). Preparatory visuo-motor cortical network of the contingent negative variation estimated by current density. Neuroimage 20, 216–224. 10.1016/s1053-8119(03)00295-7 14527582

[B68] Greimel E. Wanderer S. Rothenberger A. Herpertz-Dahlmann B. Konrad K. Roessner V. (2011). Attentional performance in children and adolescents with tic disorder and co-occurring attention-deficit/hyperactivity disorder: new insights from a 2 × 2 factorial design study. J. Abnorm. Child Psychol. 39, 819–828. 10.1007/s10802-011-9493-7 21331638 PMC3111554

[B69] Gusnard D. A. Akbudak E. Shulman G. L. Raichle M. E. (2001a). Medial prefrontal cortex and self-referential mental activity: relation to a default mode of brain function. Proc. Natl. Acad. Sci. U S A 98, 4259–4264. 10.1073/pnas.071043098 11259662 PMC31213

[B70] Gusnard D. A. Raichle M. E. Raichle M. E. (2001b). Searching for a baseline: functional imaging and the resting human brain. Nat. Rev. Neurosci. 2, 685–694. 10.1038/35094500 11584306

[B71] Harmony T. Marosi E. Becker J. Rodríguez M. Reyes A. Fernández T.. (1995). Longitudinal quantitative EEG study of children with different performances on a reading-writing test. Electroencephalogr. Clin. Neurophysiol. 95, 426–433. 10.1016/0013-4694(95)00135-2 8536571

[B72] Hasson R. Fine J. G. (2012). Gender differences among children with ADHD on continuous performance tests: a meta-analytic review. J. Atten. Disord. 16, 190–198. 10.1177/1087054711427398 22290696

[B73] Heinrich H. Busch K. Studer P. Erbe K. Moll G. H. Kratz O. (2014). EEG spectral analysis of attention in ADHD: implications for NF training? Front. Hum. Neurosci. 8:611. 10.3389/fnhum.2014.00611 25191248 PMC4139738

[B74] Heinrich H. Gevensleben H. Strehl U. (2007). Annotation: NF - train your brain to train behaviour. J. Child Psychol. Psychiatry 48, 3–16. 10.1111/j.1469-7610.2006.01665.x 17244266

[B75] Helps S. K. Broyd S. J. James C. J. Karl A. Chen W. Sonuga-Barke E. J. (2010). Altered spontaneous low frequency brain activity in attention deficit/hyperactivity disorder. Brain Res. 1322, 134–143. 10.1016/j.brainres.2010.01.057 20117101

[B76] Hennighausen K. Schulte-Körne G. Warnke A. Remschmidt H. (2000). [Contingent negative variation (CNV) in children with hyperkinetic syndrome–an experimental study using the Continuous Performance Test (CPT)]. Z. Kinder Jugendpsychiatr. Psychother. 28, 239–246. 10.1024/1422-4917.28.4.239 11103472

[B77] Houghton S. Douglas G. West J. Whiting K. Wall M. Langsford S.. (1999). Differential patterns of executive function in children with attention-deficit hyperactivity disorder according to gender and subtype. J. Child Neurol. 14, 801–805. 10.1177/088307389901401206 10614567

[B78] Hughes M. E. Johnston P. J. Fulham W. R. Budd T. W. Michie P. T. (2013). Stop-signal task difficulty and the right inferior frontal gyrus. Behav. Brain Res. 256, 205–213. 10.1016/j.bbr.2013.08.026 23973765

[B79] Ikeda A. Shibasaki H. Kaji R. Terada K. Nagamine T. Honda M.. (1997). Dissociation between contingent negative variation (CNV) and Bereitschaftspotential (BP) in patients with parkinsonism. Electroencephalogr. Clin. Neurophysiol. 102, 142–151. 10.1016/s0921-884x(96)95067-5 9060866

[B80] Jackson S. R. Parkinson A. Jung J. Ryan S. E. Morgan P. S. Hollis C.. (2011). Compensatory neural reorganization in Tourette syndrome. Curr. Biol. 21, 580–585. 10.1016/j.cub.2011.02.047 21439830 PMC3076629

[B81] John E. R. Easton P. Prichep L. Ahn H. Kaye H. Baird H. (1982). “Neurometric features of children with various cognitive functions or neurological disorders,” in Event-Related Potentials in Children, ed. Rothenberger A. (Amsterdam: Elsevier), 389–403.

[B82] Jonkman L. M. Kemner C. Verbaten M. N. Van Engeland H. Kenemans J. L. Camfferman G.. (1999). Perceptual and response interference in children with attention-deficit hyperactivity disorder and the effects of methylphenidate. Psychophysiology 36, 419–429. 10.1111/1469-8986.3640419 10432791

[B83] Kieling C. Kieling R. R. Rohde L. A. Frick P. J. Moffitt T. Nigg J. T.. (2010). The age at onset of attention deficit hyperactivity disorder. Am. J. Psychiatry 167, 14–16. 10.1176/appi.ajp.2009.09060796 20068122 PMC4478075

[B84] King S. Griffin S. Hodges Z. Weatherly H. Asseburg C. Richardson G.. (2006). A systematic review and economic model of the effectiveness and cost-effectiveness of methylphenidate, dexamfetamine and atomoxetine for the treatment of attention deficit hyperactivity disorder in children and adolescents. Health Technol. Assess. 10, iii–iv, xiii–146. 10.3310/hta10230 16796929

[B85] Klein R. G. Mannuzza S. Olazagasti M. A. Roizen E. Hutchison J. A. Lashua E. C.. (2012). Clinical and functional outcome of childhood attention-deficit/hyperactivity disorder 33 years later. Arch. Gen. Psychiatry 69, 1295–1303. 10.1001/archgenpsychiatry.2012.271 23070149 PMC3597443

[B86] Klimesch W. Doppelmayr M. Wimmer H. Gruber W. Röhm D. Schwaiger J.. (2001). Alpha and beta band power changes in normal and dyslexic children. Clin. Neurophysiol. 112, 1186–1195. 10.1016/s1388-2457(01)00543-0 11516730

[B87] Klorman R. Hazel-Fernandez L. A. Shaywitz S. E. Fletcher J. M. Marchione K. E. Holahan J. M.. (1999). Executive functioning deficits in attention-deficit/hyperactivity disorder are independent of oppositional defiant or reading disorder. J. Am. Acad. Child Adolesc. Psychiatry 38, 1148–1155. 10.1097/00004583-199909000-00020 10504814

[B88] Kok A. (1986). Effects of degradation ov visual stimuli on components of the event related potential (ERP) in Go/nogo reaction tasks. Biol. Psychol. 23, 21–38. 10.1016/0301-0511(86)90087-6 3790646

[B89] Kok A. (1999). Varieties of inhibition: manifestations in cognition, event-related potentials and aging. Acta Psychol. (Amst) 101, 129–158. 10.1016/s0001-6918(99)00003-7 10344183

[B90] Kratz O. Studer P. Baack J. Malcherek S. Erbe K. Moll G. H.. (2012). Differential effects of methylphenidate and atomoxetine on attentional processes in children with ADHD: an event-related potential study using the attention network test. Prog. Neuropsychopharmacol. Biol. Psychiatry 37, 81–89. 10.1016/j.pnpbp.2011.12.008 22227291

[B91] Lahey B. B. Pelham W. E. Loney J. Lee S. S. Willcutt E. (2005). Instability of the DSM-IV subtypes of ADHD from preschool through elementary school. Arch. Gen. Psychiatry 62, 896–902. 10.1001/archpsyc.62.8.896 16061767

[B92] Lansbergen M. M. Kenemans J. L. van Engeland H. (2007). Stroop interference and attention-deficit/hyperactivity disorder: a review and meta-analysis. Neuropsychology 21, 251–262. 10.1037/0894-4105.21.2.251 17402825

[B93] Lawrence C. A. Barry R. J. Clarke A. R. Johnstone S. J. McCarthy R. Selikowitz M.. (2005). Methylphenidate effects in attention deficit/hyperactivity disorder: electrodermal and ERP measures during a continuous performance task. Psychopharmacology (Berl) 183, 81–91. 10.1007/s00213-005-0144-y 16160877

[B94] Leckman J. F. (2002). Tourette’s syndrome. Lancet 360, 1577–1586. 10.1016/S0140-6736(02)11526-1 12443611

[B95] Leckman J. F. Vaccarino F. M. Kalanithi P. S. Rothenberger A. (2006). Annotation: Tourette syndrome: a relentless drumbeat–driven by misguided brain oscillations. J. Child Psychol. Psychiatry 47, 537–550. 10.1111/j.1469-7610.2006.01620.x 16712630

[B96] Li C. S. Yan P. Bergquist K. L. Sinha R. (2007). Greater activation of the “default” brain regions predicts stop signal errors. Neuroimage 38, 640–648. 10.1016/j.neuroimage.2007.07.021 17884586 PMC2097963

[B97] Liechti M. D. Maurizio S. Heinrich H. Jäncke L. Meier L. Steinhausen H. C.. (2012). First clinical trial of tomographic NF in attention-deficit/hyperactivity disorder: evaluation of voluntary cortical control. Clin. Neurophysiol. 123, 1989–2005. 10.1016/j.clinph.2012.03.016 22608481

[B98] Liechti M. D. Valko L. Müller U. C. Döhnert M. Drechsler R. Steinhausen H. C.. (2013). Diagnostic value of resting electroencephalogram in attention-deficit/hyperactivity disorder across the lifespan. Brain Topogr. 26, 135–151. 10.1007/s10548-012-0258-6 23053601

[B99] Lijffijt M. Kenemans J. L. Verbaten M. N. van Engeland H. (2005). A meta-analytic review of stopping performance in attention-deficit/hyperactivity disorder: deficient inhibitory motor control? J. Abnorm. Psychol. 114, 216–222. 10.1037/0021-843x.114.2.216 15869352

[B100] Linssen A. M. Vuurman E. F. Sambeth A. Nave S. Spooren W. Vargas G.. (2011). Contingent negative variation as a dopaminergic biomarker: evidence from dose-related effects of methylphenidate. Psychopharmacology (Berl) 218, 533–542. 10.1007/s00213-011-2345-x 21597989 PMC3210368

[B101] Liotti M. Pliszka S. R. Perez R. 3rd Luus B. Glahn D. Semrud-Clikeman M. (2007). Electrophysiological correlates of response inhibition in children and adolescents with ADHD: influence of gender, age and previous treatment history. Psychophysiology 44, 936–948. 10.1111/j.1469-8986.2007.00568.x 17666028

[B102] Liu Y. Miao W. Wang J. Gao P. Yin G. Zhang L.. (2013). Structural abnormalities in early Tourette syndrome children: a combined voxel-based morphometry and tract-based spatial statistics study. PLoS One 8:e76105. 10.1371/journal.pone.0076105 24098769 PMC3786886

[B103] Loeber R. Burke J. D. Lahey B. B. Winters A. Zera M. (2000). Oppositional defiant and conduct disorder: a review of the past 10 years, part I. J. Am. Acad. Child Adolesc. Psychiatry 39, 1468–1484. 10.1097/00004583-200012000-00007 11128323

[B104] Logemann H. N. Lansbergen M. M. Van Os T. W. Böcker K. B. Kenemans J. L. (2010). The effectiveness of EEG-feedback on attention, impulsivity and EEG: a sham feedback controlled study. Neurosci. Lett. 479, 49–53. 10.1016/j.neulet.2010.05.026 20478360

[B105] Lorber M. F. (2004). Psychophysiology of aggression, psychopathy and conduct problems: a meta-analysis. Psychol. Bull. 130, 531–552. 10.1037/0033-2909.130.4.531 15250812

[B106] Luman M. van Noesel S. J. Papanikolau A. Van Oostenbruggen-Scheffer J. Veugelers D. Sergeant J. A.. (2009). Inhibition, reinforcement sensitivity and temporal information processing in ADHD and ADHD+ODD: evidence of a separate entity? J. Abnorm. Child Psychol. 37, 1123–1135. 10.1007/s10802-009-9334-0 19543967 PMC2766046

[B107] Lütcke H. Gevensleben H. Albrecht B. Frahm J. (2008). Brain networks involved in early versus late response anticipation and their relation to conflict processing. J. Cogn. Neurosci. 21, 2172–2184. 10.1162/jocn.2008.21165 19016602

[B108] Macar F. Vidal F. (2003). The CNV peak: an index of decision making and temporal memory. Psychophysiology 40, 950–954. 10.1111/1469-8986.00113 14986848

[B109] Maedgen J. W. Carlson C. L. (2000). Social functioning and emotional regulation in the attention deficit hyperactivity disorder subtypes. J. Clin. Child Psychol. 29, 30–42. 10.1207/s15374424jccp29014 10693030

[B110] Mantini D. Perrucci M. G. Del Gratta C. Romani G. L. Corbetta M. (2007). Electrophysiological signatures of resting state networks in the human brain. Proc. Natl. Acad. Sci. U S A 104, 13170–13175. 10.1073/pnas.0700668104 17670949 PMC1941820

[B111] Marsh R. Zhu H. Wang Z. Skudlarski P. Peterson B. S. (2007). A developmental fMRI study of self-regulatory control in Tourette’s syndrome. Am. J. Psychiatry 164, 955–966. 10.1176/appi.ajp.164.6.955 17541057 PMC2291294

[B112] Marx A. M. Ehlis A. C. Furdea A. Holtmann M. Banaschewski T. Brandeis D.. (2015). Near-infrared spectroscopy (NIRS) NF as a treatment for children with attention deficit hyperactivity disorder (ADHD)-a pilot study. Front. Hum. Neurosci. 8:1038. 10.3389/fnhum.2014.01038 25610390 PMC4285751

[B113] Matousek M. Petersen J. (1973). “Frequency analysis of the EEG in normal children and adolescents,” in Automation of Clinical Encephalography, eds Kellaway P. Petersen J. (New York: Raven), 15–102.

[B114] Matthys W. Vanderschuren L. J. Schutter D. J. (2013). The neurobiology of oppositional defiant disorder and conduct disorder: altered functioning in three mental domains. Dev. Psychopathol. 25, 193–207. 10.1017/s0954579412000272 22800761

[B115] Mayer K. Wyckoff S. N. Strehl U. (2013). One size fits all? Slow cortical potentials NF: a review. J. Atten. Disord. 17, 393–409. 10.1177/1087054712468053 23264371

[B116] McLoughlin G. Albrecht B. Banaschewski T. Rothenberger A. Brandeis D. Asherson P.. (2009). Performance monitoring is altered in adult ADHD: a familial event-related potential investigation. Neuropsychologia 47, 3134–3142. 10.1016/j.neuropsychologia.2009.07.013 19643116 PMC3776647

[B117] McLoughlin G. Albrecht B. Banaschewski T. Rothenberger A. Brandeis D. Asherson P.. (2010). Electrophysiological evidence for abnormal preparatory states and inhibitory processing in adult ADHD. Behav. Brain Funct. 6:66. 10.1186/1744-9081-6-66 21029446 PMC2988695

[B118] McLoughlin G. Asherson P. Albrecht B. Banaschewski T. Rothenberger A. Brandeis D.. (2011). Cognitive-electrophysiological indices of attentional and inhibitory processing in adults with ADHD: familial effects. Behav. Brain Funct. 7:26. 10.1186/1744-9081-7-26 21752266 PMC3168399

[B119] Meyer M. C. Janssen R. J. Van Oort E. S. Beckmann C. F. Barth M. (2013). The quest for EEG power band correlation with ICA derived fMRI resting state networks. Front. Hum. Neurosci. 7:315. 10.3389/fnhum.2013.00315 23805098 PMC3691889

[B120] Micoulaud-Franchi J. A. Geoffroy P. A. Fond G. Lopez R. Bioulac S. Philip P. (2014). EEG NF treatments in children with ADHD: an updated meta-analysis of randomized controlled trials. Front. Hum. Neurosci. 8:906. 10.3389/fnhum.2014.00906 25431555 PMC4230047

[B121] Miller E. K. Cohen J. D. (2001). An integrative theory of prefrontal cortex function. Annu. Rev. Neurosci. 24, 167–202. 10.1146/annurev.neuro.24.1.167 11283309

[B122] Molina B. S. Hinshaw S. P. Swanson J. M. Arnold L. E. Vitiello B. Jensen P. S.. (2009). The MTA at 8 years: prospective follow-up of children treated for combined-type ADHD in a multisite study. J. Am. Acad. Child Adolesc. Psychiatry 48, 484–500. 10.1097/CHI.0b013e31819c23d0 19318991 PMC3063150

[B123] Moll G. H. Heinrich H. Gevensleben H. Rothenberger A. (2006). Tic distribution and inhibitory processes in the sensorimotor circuit during adolescence: a cross-sectional TMS study. Neurosci. Lett. 403, 96–99. 10.1016/j.neulet.2006.04.021 16690208

[B124] Moll G. H. Heinrich H. Trott G. E. Wirth S. Bock N. Rothenberger A. (2001). Children with comorbid attention-deficit-hyperactivity disorder and tic disorder: evidence for additive inhibitory deficits within the motor system. Ann. Neurol. 49, 393–396. 10.1002/ana.77.abs 11261515

[B125] Mullane J. C. Corkum P. V. Klein R. M. McLaughlin E. (2009). Interference control in children with and without ADHD: a systematic review of Flanker and Simon task performance. Child Neuropsychol. 15, 321–342. 10.1080/09297040802348028 18850349

[B126] Muller-Vahl K. R. Cath D. C. Cavanna A. E. Dehning S. Porta M. Robertson M. M.. (2011). European clinical guidelines for Tourette syndrome and other tic disorders. Part IV: deep brain stimulation. Eur. Child Adolesc. Psychiatry 20, 209–217. 10.1007/s00787-011-0166-4 21445726

[B127] Müller-Vahl K. R. Grosskreutz J. Prell T. Kaufmann J. Bodammer N. Peschel T. (2014). Tics are caused by alterations in prefrontal areas, thalamus and putamen, while changes in the cingulate gyrus reflect secondary compensatory mechanisms. BMC Neurosci 15:6. 10.1186/1471-2202-15-6 24397347 PMC3893393

[B128] Neuner I. Werner C. J. Arrubla J. Stöcker T. Ehlen C. Wegener H. P.. (2014). Imaging the where and when of tic generation and resting state networks in adult Tourette patients. Front. Hum. Neurosci. 8:362. 10.3389/fnhum.2014.00362 24904391 PMC4035756

[B129] Newcorn J. H. Halperin J. M. (2005). “Attention-deficit disorders with oppositionality and aggression,” in Attention-Deficit Disorders and Comorbidities in Children, Adolescents and Adults, ed. Brown T. E. (Washington, DC: American Psychiatric Press, Inc.), 171–207.

[B130] Nieuwenhuis S. Yeung N. van den Wildenberg W. Ridderinkhof K. R. (2003). Electrophysiological correlates of anterior cingulate function in a go/no-go task: effects of response conflict and trial type frequency. Cogn. Affect. Behav. Neurosci. 3, 17–26. 10.3758/cabn.3.1.17 12822595

[B131] Nigg J. T. Blaskey L. G. Huang-Pollock C. L. Rappley M. D. (2002). Neuropsychological executive functions and DSM-IV ADHD subtypes. J. Am. Acad. Child Adolesc. Psychiatry 41, 59–66. 10.1097/00004583-200201000-00012 11800208

[B132] Nigg J. T. Willcutt E. G. Doyle A. E. Sonuga-Barke E. J. (2005). Causal heterogeneity in attention-deficit/hyperactivity disorder: do we need neuropsychologically impaired subtypes? Biol. Psychiatry 57, 1224–1230. 10.1016/j.biopsych.2004.08.025 15949992

[B133] Orth M. (2009). Transcranial magnetic stimulation in Gilles de la Tourette syndrome. J. Psychosom. Res. 67, 591–598. 10.1016/j.jpsychores.2009.07.014 19913663

[B134] Orth M. Rothwell J. C. (2009). Motor cortex excitability and comorbidity in Gilles de la Tourette syndrome. J. Neurol. Neurosurg. Psychiatry. 80, 29–34. 10.1136/jnnp.2008.149484 18931001

[B135] Overtoom C. C. Verbaten M. N. Kemner C. Kenemans J. L. van Engeland H. Buitelaar J. K.. (1998). Associations between event-related potentials and measures of attention and inhibition in the continuous performance task in children with ADHD and normal controls. J. Am. Acad. Child Adolesc. Psychiatry 37, 977–985. 10.1097/00004583-199809000-00018 9735617

[B136] Pennington B. F. Ozonoff S. (1996). Executive functions and developmental psychopathology. J. Child Psychol. Psychiatry 37, 51–87. 10.1111/j.1469-7610.1996.tb01380.x 8655658

[B137] Perchet C. Revol O. Fourneret P. Mauguière F. Garcia-Larrea L. (2001). Attention shifts and anticipatory mechanisms in hyperactive children: an ERP study using the Posner paradigm. Biol. Psychiatry 50, 44–57. 10.1016/s0006-3223(00)01119-7 11457423

[B138] Peterson B. S. Skudlarski P. Anderson A. W. Zhang H. Gatenby J. C. Lacadie C. M.. (1998). A functional magnetic resonance imaging study of tic suppression in Tourette syndrome. Arch. Gen. Psychiatry 55, 326–333. 10.1001/archpsyc.55.4.326 9554428

[B139] Peterson B. S. Thomas P. Kane M. J. Scahill L. Zhang H. Bronen R.. (2003). Basal ganglia volumes in patients with Gilles de la Tourette syndrome. Arch. Gen. Psychiatry 60, 415–424. 10.1001/archpsyc.60.4.415 12695320

[B140] Plessen K. J. Wentzel-Larsen T. Hugdahl K. Feineigle P. Klein J. Staib L. H.. (2004). Altered interhemispheric connectivity in individuals with Tourette’s disorder. Am. J. Psychiatry 161, 2028–2037. 10.1176/appi.ajp.161.11.2028 15514403

[B141] Plichta M. M. Scheres A. (2014). Ventral-striatal responsiveness during reward anticipation in ADHD and its relation to trait impulsivity in the healthy population: a meta-analytic review of the fMRI literature. Neurosci. Biobehav. Rev. 38, 125–134. 10.1016/j.neubiorev.2013.07.012 23928090 PMC3989497

[B142] Pliszka S. R. Glahn D. C. Semrud-Clikeman M. Franklin C. Perez R. 3rd Xiong J.. (2006). Neuroimaging of inhibitory control areas in children with attention deficit hyperactivity disorder who were treatment naive or in long-term treatment. Am. J. Psychiatry 163, 1052–1060. 10.1176/appi.ajp.163.6.1052 16741206

[B143] Pliszka S. R. Liotti M. Woldorff M. G. (2000). Inhibitory control in children with attention-deficit/hyperactivity disorder: event-related potentials identify the processing component and timing of an impaired right-frontal response-inhibition mechanism. Biol. Psychiatry 48, 238–246. 10.1016/s0006-3223(00)00890-8 10924667

[B144] Polanczyk G. Rohde L. A. (2007). Epidemiology of attention-deficit/hyperactivity disorder across the lifespan. Curr. Opin. Psychiatry 20, 386–392. 10.1097/yco.0b013e3281568d7a 17551354

[B145] Praamstra P. Pope P. (2007). Slow brain potential and oscillatory EEG manifestations of impaired temporal preparation in Parkinson’s disease. J. Neurophysiol. 98, 2848–2857. 10.1152/jn.00224.2007 17728390

[B146] Pulvermüller F. Lutzenberger W. Müller V. Mohr B. Dichgans J. Birbaumer N. (1996). P3 and contingent negative variation in Parkinson’s disease. Electroencephalogr. Clin. Neurophysiol. 98, 456–467. 10.1016/0013-4694(96)95537-6 8763505

[B147] Raine A. (2002). Biosocial studies of antisocial and violent behavior in children and adults: a review. J. Abnorm. Child Psychol. 30, 311–326. 10.1023/A:1015754122318 12108763

[B148] Rockstroh B. Elbert T. Lutzenberger W. Birbaumer N. (1984). “Operant control of slow brain potentials: a tool in the investigation of the potential’s meaning and its relation to attentional dysfunction,” in Self-Regulation of the Brain and Behaviour, eds Elbert T. Rockstroh B. Lutzenberger W. Birbaumer N. (Heidelberg: Springer), 227–239.

[B149] Roessner V. Albrecht B. Dechent P. Baudewig J. Rothenberger A. (2008). Normal response inhibition in boys with Tourette syndrome. Behav. Brain Funct. 4:29. 10.1186/1744-9081-4-29 18638368 PMC2491645

[B150] Roessner V. Becker A. Banaschewski T. Rothenberger A. (2007). Psychopathological profile in children with chronic tic disorder and co-existing ADHD: additive effects. J. Abnorm. Child Psychol. 35, 79–85. 10.1007/s10802-006-9086-z 17171537

[B151] Roessner V. Overlack S. Baudewig J. Dechent P. Rothenberger A. Helms G. (2009). No brain structure abnormalities in boys with Tourette’s syndrome: a voxel-based morphometry study. Mov. Disord. 24, 2398–2403. 10.1002/mds.22847 19890999

[B152] Roessner V. Plessen K. J. Rothenberger A. Ludolph A. G. Rizzo R. Skov L.. (2011). European clinical guidelines for Tourette syndrome and other tic disorders. Part II: pharmacological treatment. Eur. Child Adolesc. Psychiatry 20, 173–196. 10.1007/s00787-011-0163-7 21445724 PMC3065650

[B153] Rothenberger A. (2009). Brain oscillations forever–neurophysiology in future research of child psychiatric problems. J. Child Psychol. Psychiatry 50, 79–86. 10.1111/j.1469-7610.2008.01994.x 19220591

[B154] Rothenberger A. Gevensleben H. (2013). “Selbstregulation von Tics—Optimierung durch NF,” in NF: Ein Praxisbuch, ed. Strehl U. (Stuttgart: Kohlhammer), 136–148.

[B155] Rubia K. (2011). “Cool” inferior frontostriatal dysfunction in attention-deficit/hyperactivity disorder versus “hot” ventromedial orbitofrontal-limbic dysfunction in conduct disorder: a review. Biol. Psychiatry 69, e69–e87. 10.1016/j.biopsych.2010.09.023 21094938

[B156] Rubia K. Halari R. Smith A. B. Mohammad M. Scott S. Brammer M. J. (2009a). Shared and disorder-specific prefrontal abnormalities in boys with pure attention-deficit/hyperactivity disorder compared to boys with pure CD during interference inhibition and attention allocation. J. Child Psychol. Psychiatry 50, 669–678. 10.1111/j.1469-7610.2008.02022.x 19236528

[B157] Rubia K. Halari R. Smith A. B. Mohammed M. Scott S. Giampietro V.. (2008). Dissociated functional brain abnormalities of inhibition in boys with pure conduct disorder and in boys with pure attention deficit hyperactivity disorder. Am. J. Psychiatry 165, 889–897. 10.1176/appi.ajp.2008.07071084 18413706

[B158] Rubia K. Overmeyer S. Taylor E. Brammer M. Williams S. C. Simmons A.. (1999). Hypofrontality in attention deficit hyperactivity disorder during higher-order motor control: a study with functional MRI. Am. J. Psychiatry 156, 891–896. 10.1176/ajp.156.6.891 10360128

[B159] Rubia K. Smith A. B. Halari R. Matsukura F. Mohammad M. Taylor E.. (2009b). Disorder-specific dissociation of orbitofrontal dysfunction in boys with pure conduct disorder during reward and ventrolateral prefrontal dysfunction in boys with pure ADHD during sustained attention. Am. J. Psychiatry 166, 83–94. 10.1176/appi.ajp.2008.08020212 18829871

[B160] Rucklidge J. J. Tannock R. (2002). Neuropsychological profiles of adolescents with ADHD: effects of reading difficulties and gender. J. Child Psychol. Psychiatry 43, 988–1003. 10.1111/1469-7610.00227 12455921

[B161] Sagvolden T. Aase H. Zeiner P. Berger D. (1998). Altered reinforcement mechanisms in attention-deficit/hyperactivity disorder. Behav. Brain Res. 94, 61–71. 10.1016/s0166-4328(97)00170-8 9708840

[B162] Sagvolden T. Johansen E. B. Aase H. Russell V. A. (2005). A dynamic developmental theory of attention-deficit/hyperactivity disorder (ADHD) predominantly hyperactive/impulsive and combined subtypes. Behav. Brain Sci. 28, 397–419; discussion 419–368. 10.1017/s0140525x05000075 16209748

[B163] Schuerholz L. J. Singer H. S. Denckla M. B. (1998). Gender study of neuropsychological and neuromotor function in children with Tourette syndrome with and without attention-deficit hyperactivity disorder. J. Child Neurol. 13, 277–282. 10.1177/088307389801300607 9660511

[B164] Schwartz K. Verhaeghen P. (2008). ADHD and stroop interference from age 9 to age 41 years: a meta-analysis of developmental effects. Psychol. Med. 38, 1607–1616. 10.1017/s003329170700267x 18226285

[B165] Seidman L. J. Biederman J. Monuteaux M. C. Valera E. Doyle A. E. Faraone S. V. (2005). Impact of gender and age on executive functioning: do girls and boys with and without attention deficit hyperactivity disorder differ neuropsychologically in preteen and teenage years? Dev. Neuropsychol. 27, 79–105. 10.1207/s15326942dn27014 15737943

[B166] Seidman L. J. Biederman J. Valera E. M. Monuteaux M. C. Doyle A. E. Faraone S. V. (2006). Neuropsychological functioning in girls with attention-deficit/hyperactivity disorder with and without learning disabilities. Neuropsychology 20, 166–177. 10.1037/0894-4105.20.2.166 16594777

[B167] Sergeant J. (2000). The cognitive-energetic model: an empirical approach to attention-deficit hyperactivity disorder. Neurosci. Biobehav. Rev. 24, 7–12. 10.1016/s0149-7634(99)00060-3 10654654

[B168] Sharp W. S. Walter J. M. Marsh W. L. Ritchie G. F. Hamburger S. D. Castellanos F. X. (1999). ADHD in girls: clinical comparability of a research sample. J. Am. Acad. Child Adolesc. Psychiatry 38, 40–47. 10.1097/00004583-199901000-00018 9893415

[B169] Siniatchkin M. Kuppe A. (2011). Neurophysiological determinants of tic severity in children with chronic motor tic disorder. Appl. Psychophysiol. Biofeedback 36, 121–127. 10.1007/s10484-011-9155-0 21431421

[B170] Smith A. B. Taylor E. Brammer M. Halari R. Rubia K. (2008). Reduced activation in right lateral prefrontal cortex and anterior cingulate gyrus in medication-naive adolescents with attention deficit hyperactivity disorder during time discrimination. J. Child Psychol. Psychiatry 49, 977–985. 10.1111/j.1469-7610.2008.01870.x 18759938

[B171] Sonuga-Barke E. J. (2005). Causal models of attention-deficit/hyperactivity disorder: from common simple deficits to multiple developmental pathways. Biol. Psychiatry 57, 1231–1238. 10.1016/j.biopsych.2004.09.008 15949993

[B172] Sonuga-Barke E. J. Castellanos F. X. (2007). Spontaneous attentional fluctuations in impaired states and pathological conditions: a neurobiological hypothesis. Neurosci. Biobehav. Rev. 31, 977–986. 10.1016/j.neubiorev.2007.02.005 17445893

[B173] Sonuga-Barke E. J. Brandeis D. Cortese S. Daley D. Ferrin M. Holtmann M.. (2013). Nonpharmacological interventions for ADHD: systematic review and meta-analyses of randomized controlled trials of dietary and psychological treatments. Am. J. Psychiatry 170, 275–289. 10.1176/appi.ajp.2012.12070991 23360949

[B174] Strehl U. Leins U. Goth G. Klinger C. Hinterberger T. Birbaumer N. (2006). Self-regulation of slow cortical potentials: a new treatment for children with attention-deficit/hyperactivity disorder. Pediatrics 118, e1530–e1540. 10.1542/peds.2005-2478 17060480

[B175] Suri R. E. Schultz W. (2001). Temporal difference model reproduces anticipatory neural activity. Neural Comput. 13, 841–862. 10.1162/089976601300014376 11255572

[B176] Swain J. E. Scahill L. Lombroso P. J. King R. A. Leckman J. F. (2007). Tourette syndrome and tic disorders: a decade of progress. J. Am. Acad. Child Adolesc. Psychiatry 46, 947–968. 10.1097/chi.0b013e318068fbcc 17667475

[B177] Swanson J. Arnold L. E. Kraemer H. Hechtman L. Molina B. Hinshaw S.. (2008). Evidence, interpretation and qualification from multiple reports of long-term outcomes in the multimodal treatment study of children with ADHD (MTA): part I: executive summary. J. Atten. Disord. 12, 4–14. 10.1177/1087054708319345 18573923

[B178] Tannock R. (1998). Attention deficit hyperactivity disorder: advances in cognitive, neurobiological and genetic research. J. Child Psychol. Psychiatry 39, 65–99. 10.1017/s0021963097001777 9534087

[B179] Tansey M. A. (1986). A simple and a complex tic (Gilles de la Tourette’s syndrome): their response to EEG sensorimotor rhythm biofeedback training. Int. J. Psychophysiol. 4, 91–97. 10.1016/0167-8760(86)90002-4 3460976

[B180] Taylor E. Döpfner M. Sergeant J. Asherson P. Banaschewski T. Buitelaar J.. (2004). European clinical guidelines for hyperkinetic disorder – first upgrade. Eur. Child Adolesc. Psychiatry 13(Suppl. 1), I7–I30. 10.1007/s00787-004-1002-x 15322953

[B181] Tripp G. Wickens J. R. (2008). Research review: dopamine transfer deficit: a neurobiological theory of altered reinforcement mechanisms in ADHD. J. Child Psychol. Psychiatry 49, 691–704. 10.1111/j.1469-7610.2007.01851.x 18081766

[B182] Uddin L. Q. Kelly A. M. Biswal B. B. Margulies D. S. Shehzad Z. Shaw D.. (2008). Network homogeneity reveals decreased integrity of default-mode network in ADHD. J. Neurosci. Methods 169, 249–254. 10.1016/j.jneumeth.2007.11.031 18190970

[B183] van de Loo-Neus G. H. Rommelse N. Buitelaar J. K. (2011). To stop or not to stop? How long should medication treatment of attention-deficit hyperactivity disorder be extended? Eur. Neuropsychopharmacol. 21, 584–599. 10.1016/j.euroneuro.2011.03.008 21530185

[B184] van Goozen S. H. Fairchild G. Snoek H. Harold G. T. (2007). The evidence for a neurobiological model of childhood antisocial behavior. Psychol. Bull. 133, 149–182. 10.1037/0033-2909.133.1.149 17201574

[B185] van Leeuwen T. H. Steinhausen H. C. Overtoom C. C. Pascual-Marqui R. D. Van’t Klooster B. Rothenberger A.. (1998). The continuous performance test revisited with neuroelectric mapping: impaired orienting in children with attention deficits. Behav. Brain Res. 94, 97–110. 10.1016/s0166-4328(97)00173-3 9708843

[B186] van Mourik R. Oosterlaan J. Sergeant J. A. (2005). The stroop revisited: a meta-analysis of interference control in AD/HD. J. Child Psychol. Psychiatry 46, 150–165. 10.1111/j.1469-7610.2004.00345.x 15679524

[B187] van Mourik R. Papanikolau A. Van Gellicum-Bijlhout J. Van Oostenbruggen J. Veugelers D. Post-Uiterweer A.. (2009). Interference control in children with attention deficit/hyperactivity disorder. J. Abnorm. Child Psychol. 37, 293–303. 10.1007/s10802-008-9277-x 18931905

[B188] Van Veen V. Carter C. S. (2002). The timing of action-monitoring processes in the anterior cingulate cortex. J. Cogn. Neurosci. 14, 593–602. 10.1162/08989290260045837 12126500

[B189] Verdellen C. Van De Griendt J. Hartmann A. Murphy T. Group E. G. (2011). European clinical guidelines for Tourette syndrome and other tic disorders. Part III: behavioural and psychosocial interventions. Eur. Child Adolesc. Psychiatry 20, 197–207. 10.1007/s00787-011-0167-3 21445725

[B190] Vloet T. D. Neufang S. Herpertz-Dahlmann B. Konrad K. (2006). [Neuroimaging data of ADHD, tic-disorder and obsessive-compulsive-disorder in children and adolescents]. Z. Kinder Jugendpsychiatr. Psychother. 34, 343–355. 10.1024/1422-4917.34.5.343 16981155

[B191] Vollebregt M. A. Van Dongen-Boomsma M. Buitelaar J. K. Slaats-Willemse D. (2014). Does EEG-NF improve neurocognitive functioning in children with attention-deficit/hyperactivity disorder? A systematic review and a double-blind placebo-controlled study. J. Child Psychol. Psychiatry 55, 460–472. 10.1111/jcpp.12143 24168522

[B192] Walter W. G. Cooper R. Aldridge V. J. Mccallum W. C. Winter A. L. (1964). Contingent negative variation: an electric sign of sensorimotor association and expectancy in the human brain. Nature 203, 380–384. 10.1038/203380a0 14197376

[B193] Wang J. R. Hsieh S. (2013). NF training improves attention and working memory performance. Clin. Neurophysiol. 124, 2406–2420. 10.1016/j.clinph.2013.05.020 23827814

[B194] Wang Z. Maia T. V. Marsh R. Colibazzi T. Gerber A. Peterson B. S. (2011). The neural circuits that generate tics in Tourette’s syndrome. Am. J. Psychiatry 168, 1326–1337. 10.1176/appi.ajp.2011.09111692 21955933 PMC4246702

[B195] Weissman D. H. Roberts K. C. Visscher K. M. Woldorff M. G. (2006). The neural bases of momentary lapses in attention. Nat. Neurosci. 9, 971–978. 10.1038/nn1727 16767087

[B196] WHO (1993). The ICD-10 Classifications of Mental and Behavioral Disorders: Clinical Descriptions and Diagnostic Guidelines 1992; Diagnostic Criteria for Research 1993. Geneva: World Health Organization.

[B197] Wiersema R. Van Der Meere J. Antrop I. Roeyers H. (2006). State regulation in adult ADHD: an event-related potential study. J. Clin. Exp. Neuropsychol. 28, 1113–1126. 10.1080/13803390500212896 16840239

[B198] Yang P. Jong Y. J. Chung L. C. Chen C. S. (2004). Gender differences in a clinic-referred sample of Taiwanese attention-deficit/hyperactivity disorder children. Psychiatry Clin. Neurosci. 58, 619–623. 10.1111/j.1440-1819.2004.01312.x 15601386

[B199] Yong-Liang G. Robaey P. Karayanidis F. Bourassa M. Pelletier G. Geoffroy G. (2000). ERPs and behavioral inhibition in a Go/No-go task in children with attention-deficit hyperactivity disorder. Brain Cogn. 43, 215–220. 10857697

[B200] Yordanova J. Dumais-Huber C. Rothenberger A. (1996). Coexistence of tics and hyperactivity in children: no additive at the psychophysiological level. Int. J. Psychophysiol. 21, 121–133. 10.1016/0167-8760(95)00045-3 8792201

[B201] Yordanova J. Dumais-Huber C. Rothenberger A. Woerner W. (1997). Frontocortical activity in children with comorbidity of tic disorder and attention-deficit hyperactivity disorder. Biol. Psychiatry 41, 585–594. 10.1016/s0006-3223(96)00096-0 9046991

[B202] Yordanova J. Falkenstein M. Hohnsbein J. Kolev V. (2004). Parallel systems of error processing in the brain. Neuroimage 22, 590–602. 10.1016/j.neuroimage.2004.01.040 15193587

[B203] Zavala B. A. Tan H. Little S. Ashkan K. Hariz M. Foltynie T.. (2014). Midline frontal cortex low-frequency activity drives subthalamic nucleus oscillations during conflict. J. Neurosci. 34, 7322–7333. 10.1523/jneurosci.1169-14.2014 24849364 PMC4028502

[B204] Ziemann U. Paulus W. Rothenberger A. (1997). Decreased motor inhibition in Tourette’s disorder: evidence from transcranial magnetic stimulation. Am. J. Psychiatry 154, 1277–1284. 10.1176/ajp.154.9.1277 9286189

[B205] Zuckerman M. (1994). Behavioral Expressions and Biosocial Bases of Sensation Seeking. Cambridge: Cambridge University Press.

